# Recent Advances in Desmoid Tumor Therapy

**DOI:** 10.3390/cancers12082135

**Published:** 2020-08-01

**Authors:** Andrea Napolitano, Alessandro Mazzocca, Mariella Spalato Ceruso, Alessandro Minelli, Francesca Baldo, Giuseppe Badalamenti, Marianna Silletta, Daniele Santini, Giuseppe Tonini, Lorena Incorvaia, Bruno Vincenzi

**Affiliations:** 1 Department of Medical Oncology, Università Campus Bio-Medico, 00128 Rome, Italy; a.napolitano@unicampus.it (A.N.); a.mazzocca@unicampus.it (A.M.); m.spalatoceruso@unicampus.it (M.S.C.); a.minelli@unicampus.it (A.M.); francesca.baldo.tr@gmail.com (F.B.); m.silletta@unicampus.it (M.S.); d.santini@unicampus.it (D.S.); g.tonini@unicampus.it (G.T.); 2 Department of Medical Oncology, Policlinico “Paolo Giaccone”, 90127 Palermo, Italy; giuseppe.badalamenti@unipa.it (G.B.); lorena.incorvaia@unipa.it (L.I.)

**Keywords:** desmoid tumor, aggressive fibromatosis, active surveillance, chemotherapy, tyrosine kinase inhibitors

## Abstract

The desmoid tumor is a locally aggressive proliferative disease within the family of soft-tissue sarcomas. Despite its relatively good prognosis, the clinical management of desmoid tumors requires constant multidisciplinary evaluation due to its highly variable clinical behavior. Recently, active surveillance has being regarded as the appropriate strategy at diagnosis, as indolent persistence or spontaneous regressions are not uncommon. Here, we review the most recent advances in desmoid tumor therapy, including low-dose chemotherapy and treatment with tyrosine kinase inhibitors. We also explore the recent improvements in our knowledge of the molecular biology of this disease, which are leading to clinical trials with targeted agents.

## 1. Introduction

Desmoid tumor(s) (DT)—also known as desmoid-type fibromatosis—is a monoclonal, non-metastasizing, locally aggressive, often multifocal, fibroblastic proliferative disease within the family of soft-tissue sarcomas [1]. The incidence of DT is low, with about 2–4 new diagnoses per million individuals per year. DT more frequently affect young adults, with a peak age around 35 years, mainly women at reproductive age. They can arise in any body district: they are more commonly extra-abdominal (in the abdominal wall, limbs and girdles); less frequently, they are found intra-abdominally, often in the mesentery [2].

Based on the etiology, two main categories of DT are recognized: sporadic DT and familial adenomatous polyposis (FAP)-associated DT. Sporadic DT represent 85–90% of the total diagnoses and in this population, a striking female predominance is observed (male/female ratio of approximately 0.5). DT are diagnosed in about 10–15% of patients affected by FAP syndrome, a risk 800- to 1000-fold higher than in the general population [3]. FAP is a hereditary cancer syndrome caused by germline mutations in the *APC* (adenomatous polyposis coli) gene predisposing to hundreds of adenomatous polyps of the colon and colorectal adenocarcinoma as well as, to a lesser extent, to other cancer types [4]. Sporadic and FAP-associated DT share morphologic and biologic characteristics, although FAP-associated DT are largely more often intra-abdominal compared to sporadic DT [5].

## 2. Molecular Genetics of DT

DT are a remarkable example of tumors driven by specific molecular and genetic alterations. As a rare pathology, they are more difficult to study than more prevalent diseases. A better understanding of these alterations has finally led in the last years to the development of the first tailored therapies for DT, which are expected to revolutionize its treatment [6].

### 2.1. Canonical Wnt Pathway

**Figure 1:**
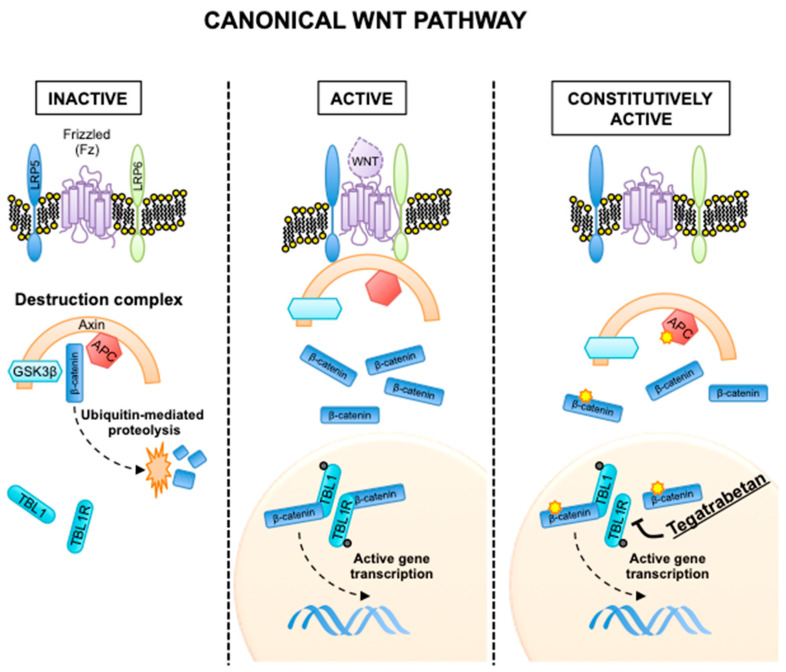
Canonical Wnt pathway in its inactive, active and constitutively active states. In the constitutively active state, the yellow stars indicate activating mutations in the β-catenin gene (*CTNNB1*) and inactivating mutations in APC. Mechanism of action of tegatrabetan is shown.

Sporadic and FAP-associated DT present alterations in the same pathway—the canonical Wnt pathway. The Wnt-signaling pathways are evolutionary conserved pathways that physiologically controls gene expression programs fundamental for embryonal development [7,8]. They are divided in canonical and non-canonical based on whether the final step regulating gene transcription is the nuclear translocation of β-catenin. In the canonical Wnt pathway, APC and other proteins form a destruction complex that tightly regulate β-catenin degradation. The activation of the pathway due to the binding of Wnt ligands to the Fz/LRP5/6 co-receptor group results in the disassembly of the destruction complex, β-catenin accumulation and nuclear translocation [7,8]. To form a transcriptionally active complex, β-catenin binds to SUMOylated transducin-β-like 1 (TBL1) and its related protein TBLR1 [9], thus resulting in the activation of genes associated with cell proliferation (Figure 1) [7,8,9].

In sporadic DT, recurrent mutations in one of three major hotspots in the exon 3 (T41A, S45F and S45P) of the β-catenin gene (*CTNNB1*) disrupt the interaction between β-catenin and APC [10]. In FAP-associated DT, inactivating mutations in APC with subsequent loss of the wild-type allele results in the same defective interaction. This ultimately results in a slower degradation of β-catenin and gene transcription independently of the presence of Wnt ligands (Figure 1). Importantly, modern sequencing technologies have shown *CTNNB1* or *APC* mutations to be near universal in DT; excluding the existence of a subgroup of “wild-type” DT encompassing about 15% of all DT cases, as proposed in the past. Other genomic events associated with Wnt activation (such as chromosome 6-loss or BMI1 mutation) can be found in the 5% of DT truly wild-type for *CTNNB1* and *APC* mutations [10,11].

Finally, β-catenin is also highly expressed in the proliferative phase of wound healing and this could partially explain the association between DT and trauma or surgical incisions, as shown also in genetically engineered mouse models [12].

### 2.2. Notch Pathway

Although alterations in the canonical Wnt pathway are driver events in the development of DT, recent studies point to an essential role also of the Notch-signaling pathway, another fundamental pathway regulating of embryonic development.

**Figure 2:**
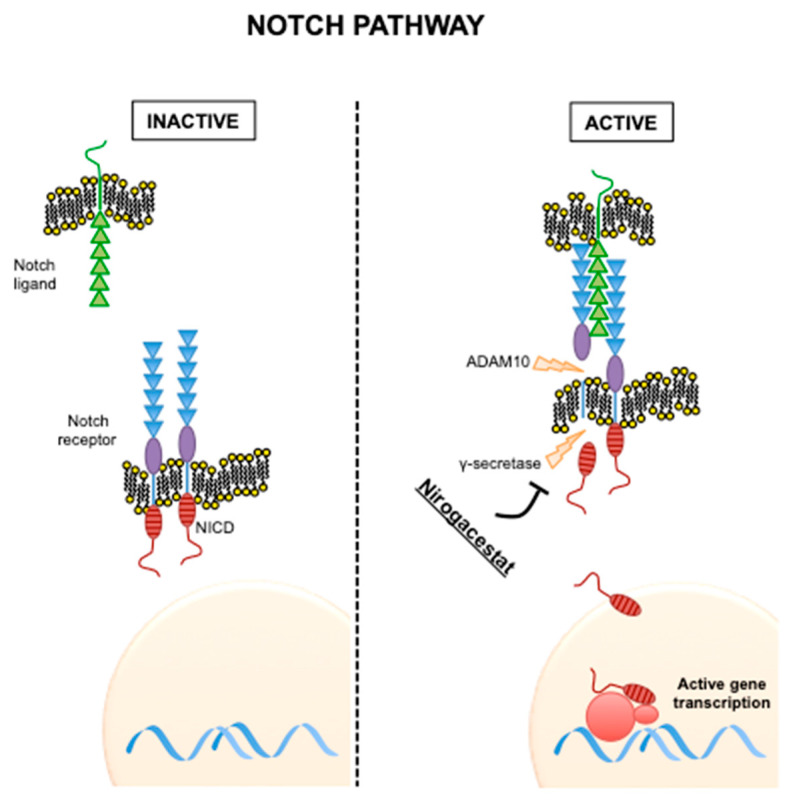
Notch pathway in its inactive and active states. Mechanism of action of nirogacestat is shown.

After ligand binding, two proteolytic cleavages in the Notch receptors occur; one in the extracellular domain by the metalloprotease ADAM10 and one in the intracellular domain by γ-secretase. This last cleavage results in the release of Notch intracellular domain (NICD). NICD is then translocated to the nucleus and activates transcriptional factors (Figure 2) [13].

Crosstalk between the Wnt and Notch pathways were first observed in *Drosophila* [14] and then confirmed in mammalian cells, in particular in colorectal cancer models [15,16]. Notably, DT were shown to highly express NOTCH1 and its downstream transcription factor HES1 [17]. This is particularly important, as inhibitors of the Notch pathway are currently in clinical development and preclinical studies support their activity in DT [17].

## 3. General Therapeutic Strategy

**Figure 3:**
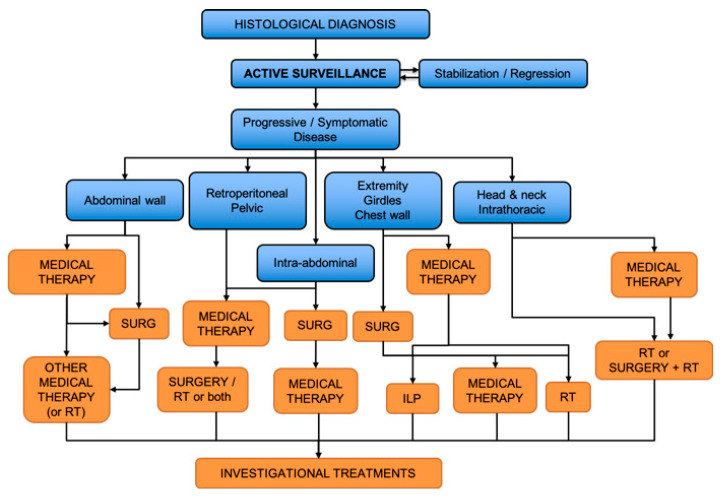
Treatment algorithm for desmoid tumors. SURG—surgery; RT—radiotherapy; ILP—isolated limb perfusion. Adapted from [2].

The therapeutic approach in DT patients requires a multidisciplinary evaluation accounting for the large variability in tumor location, extension, intrinsic aggressiveness, as well as patient symptoms and preferences. In the last years, cooperative efforts regarding consensus treatment recommendations and evidence-based guidelines were coordinated by the Desmoid Tumor Working Group. A common therapeutic algorithm derived from their efforts is shown in Figure 3 [2].

### 3.1. Active Surveillance

Until 2000, primary surgical resection was the standard of care in DT. Initially, an active surveillance was reserved to patients with recurrent disease, in order to avoid morbidity related to a second surgery or radiation therapy [18]. In the last two decades, the validity of active surveillance as a frontline approach in patients with resectable disease has also been shown [19,20,21]. In 2008, a retrospective Italian/French study showed a 50% progression-free survival (PFS) at 5 years for patients managed with active surveillance as first step [20]. Importantly, an initial observation helps to discriminate aggressive tumors from indolent lesions. Spontaneous regressions can be observed in 20–30% of cases and supports observation as first-line management. Although not fully understood, this phenomenon may at least in part be related to DT immunologic environment; in fact these tumors usually show strong immune infiltration at the tumor margins without programmed death-ligand 1 (PD-L1)-driven immune suppression [22]. Currently, a conservative approach can be considered the treatment of choice in asymptomatic or minimally symptomatic patients or those with tumors involving critical sites, as the mesentery [2]. It is still a matter of discussion whether active surveillance may be preferred in DT with specific *CTNNB1* genotypes associated with higher recurrence rates after surgery [23].

### 3.2. Surgery

The role of surgery in treatment of DT is still under debate. Several retrospective case series reporting a local control rate after surgical resection with negative margins of ~80% at five years. However, due to the peculiar infiltrative growth of these neoplasms, large resections are often required to obtain negative margins, with potential functional and cosmetic alterations.

The strongest factors predicting DT recurrence after surgery are represented by tumor size and site and patient age [24,25,26]. Among sites, abdominal DT have decreased recurrence rates after surgery compared to extra-abdominal locations [24,25,26]. The impact of microscopic margin positivity on tumor relapse is not clear, as it was not found to be predictor of a worse local control in some series [24,25,27,28], while being associated in others [19,29]. As DT are extremely infiltrative locally, the pathologic evaluation of margin negativity is particularly difficult and this may explain this unclear association. Interestingly, the specific *CTNNB1* mutation S45F also seems to be associated with a worse recurrence-free survival after surgery, although the molecular mechanisms behind these association are unclear [23,30].

Currently, surgery is considered a valid option for the local control of DT after failure of active surveillance. Many authors suggest surgical resection as therapeutic strategy if negative margins can be achieved without important functional or cosmetic sacrifice, in particular in small symptomatic cases [31]. 

### 3.3. Radiotherapy

Radiation therapy is often considered in selected cases as a salvage therapy when other therapeutic options fail. In retrospective series when surgery was still considered the standard therapy, radiotherapy alone or combined with surgery was associated with a better control rate than surgery alone [32]. In those cases when radiation therapy is selected, the recommended dose is 50–56 Gy in 28 once-daily fractions of 2 Gy [33], with higher doses associated with an increased risk of complications without improved local control [34]. The neoadjuvant use of radiation therapy may reduce the local recurrence of DT, but there are insufficient data to recommend it as standard of care [35].

### 3.4. Other Local Treatments

Other loco–regional treatments can be considered for patients with advanced DF in which surgical resection would result in significant functional impairment. Isolated limb perfusion with tumor necrosis-factor α and melphalan appears to be an effective alternative therapeutic strategy to obtain local control, especially in patients with multifocal disease involving hand and foot [36]. Cryoablation can also be potentially useful for patients with small and moderately sized extra-abdominal DT, although further studies are needed [37].

## 4. Medical Therapy

When local treatments fail or are contraindicated, several medical strategies can be considered. Systemic therapy options include nonsteroidal anti-inflammatory drugs (NSAIDs), hormonal therapy, cytotoxic chemotherapy (single agent or combinations) and target therapy. First-line medical treatment should be represented by the least toxic options (NSAIDs and hormonal therapy), while a more aggressive approach (such as combination chemotherapy) should be reserved for patient with rapidly growing and symptomatic unresectable or advanced diseases.

### 4.1. NSAIDs

Stabilization of β-catenin leads its cytoplasmic accumulation and translocation into the nucleus, where it enhances the transcription of target genes, among which *PTGS2*, the gene encoding for cyclooxygenase-2 (COX-2). Overexpression of COX-2 results in increased expression of platelet-derived growth factors (PDGFs), which contribute to tumorigenesis by stimulating angiogenesis, invasiveness and resistance to apoptosis. COX-2 expression is elevated in several tumors, including DT [38,39]. DT represent one of the first models showing nonsteroidal anti-inflammatory drugs (NSAIDs) antitumor activity, since the description of a sternal DT regression during indomethacin treatment in a patient affected by pericarditis in 1980 [40].

The molecular mechanisms of action of NSAIDs in DT are poorly understood. It is known that most NSAIDs have COX-2 dependent and independent antitumor activities [41]. The Wnt/β-catenin pathway has been suggested as a COX-2 independent target of NSAIDs [42,43]. Although the studies for NSAIDs use in DT do not derive from controlled trials, both preclinical data and clinical reports support a potential benefit of these compounds, with a good profile of tolerability [44,45].

No data have been so far specifically developed in FAP-associated DT patients. FAP patients represent an unique population of interest, as NSAIDs have been studied as potential chemo-preventative agents for FAP-associated polyps [46,47,48].

Given that spontaneous regressions are observed in absence of treatment, it is particularly difficult to establish the real benefit in disease control derived from NSAIDs.

### 4.2. Hormonal Therapy

DT—in particular, those of the abdominal wall—are frequently associated with pregnancy in women. This has been linked to the stretching trauma of the abdominal wall and to hormonal changes. Moreover, anecdotal reports of DT association to exogenous estrogen treatment or spontaneous regression during menopause are present. DT have a nearly uniform expression of estrogen receptor β (ERβ), which provides a biologic mechanism for the action of anti-estrogenic compounds in the treatment of fibromatosis [49].

Anti-hormonal agents have been investigated alone or in combination with anti-COX2 as first-line medical treatment, because of their limited toxicity. Among these, the most widely tested are tamoxifen and toremifene [50,51]. In particular, tamoxifen is associated with clinical benefit in about 30% of the cases, with most patients with symptomatic benefit not showing significant radiological changes [52]. Importantly, the use of tamoxifen in association to the NSAID sulindac has also been explored for pediatric DT patients, without significant side effects, but with limited responses [53].

Mechanistically, the antiproliferative actions of tamoxifen and toremifene have been shown to involve the modulation of transforming growth factor-β (TGF-β) and its receptors, a critical pathway regulating fibroblast proliferation [54,55].

### 4.3. Standard Chemotherapy

Conventional-dose chemotherapy is an option in those cases in which more rapid response is needed (e.g., for intra-abdominal or head and neck DT). Anthracycline-based regimes are used and are associated with a response rate of about 50% [56]. The combination regimen of doxorubicin with dacarbazine has been particularly shown to be effective and safe, although in a limited set of patients with DT unresponsive to conventional hormone therapy [57]. Pegylated liposomal doxorubicin has been reported to have significant activity with an acceptable toxicity profile, with less cardiac toxicity than conventional doxorubicin [58,59] and can therefore be considered an alternative to doxorubicin especially in young patients. Other anthracycline-free chemotherapeutic regimens used for DT patients include mitomycin and ifosfamide and etoposide, although the studies of efficacy are very limited [60].

### 4.4. Low-Dose Chemotherapy

Given the long life expectancy of most DT patients and the cumulative toxicity of anthracyclines, low-dose chemotherapy regimen based on the association of methotrexate and a vinca alkaloid (vinorelbine or vinblastine) have also been investigated.

This combination has shown prolonged activity in multiple studies and case series, with clinical and radiological benefit in more than 80% of the patients, regardless of the *CTNNB1* mutation status [61,62,63,64,65,66], with responses and prolonged clinical benefit also in the pediatric population [67]. Overall, the combination of methotrexate and a vinca alkaloid has been associated with radiological responses in about 50% of the treated patients, with responses lasting on average more than five years [61,62,63,64,65,66].

In particular, we recently showed for the first time that low-dose chemotherapy is effective also in FAP-associated DT, with a median PFS of 6.5 years in this rare population [68].

Low-dose methotrexate in combination with a vinca alkaloid currently represent a preferable alternative to full-dose chemotherapy. This regimen has indeed been used as the control group in a recent study of the tyrosine kinase inhibitor (TKI) pazopanib.

### 4.5. Targeted Therapy with TKIs

Many targeted drugs have been investigated for treatment of DF, based on the presumed role of soluble factors such as PDGFs and vascular endothelial growth factor (VEGF) in DT initiation and progression. In this respect, TKIs inhibiting the receptors of these soluble factors represent the most important and promising class of drugs.

**Imatinib.** Imatinib was the first TKI evaluated for treatment in patients with progressive DT, with a disease response rate of 10–15% and a disease control rate ranging from about 40% to 70% at six months [69,70]. Interestingly, in one of the studies a drop of serum values of PDGFR-B was observed in responding patients [69]. Moreover, imatinib appears to be particularly effective in patients with the S45F mutation of *CTNNB1* [71]. Notably, some of the patients progressing under imatinib benefited from treatment with the related TKI nilotinib [72].

**Sorafenib.** Sorafenib is another multi-target inhibitor of tyrosine kinase receptors, including VEGFRs and PDGFRs, with proven activity in the treatment of DT. In a retrospective cohort of 26 patients, the response rate was 25% (higher than imatinib) and the disease stabilization rate was 70% [73]. In 2018, the results of ALLIANCE, a phase III randomized, placebo-controlled trial of sorafenib (400 mg daily), have been published. The 2-year PFS was 81% in the Sorafenib group and 36% in the placebo group with an objective response rate of 33% and 20%, respectively [74].

**Pazopanib.** The DESMOPAZ (NCT01876082) is a randomized phase II study investigating pazopanib versus IV methotrexate/vinblastine (MV) in adult patients with progressive DT. The study showed a 6-month disease control rate of 83.7% for the pazopanib group and 45.0% for the methotrexate-vinblastine group with an objective response rate of 37% and 55%, respectively [75].

## 5. Ongoing Trials and Future Directions

Several trials investigating novel targeted therapies in advanced, unresectable DT are currently ongoing, based on our better understanding of the molecular biology underlying DT growth.

**Gamma-secretase inhibitors (GSIs).** Originally developed as anti-Alzheimer agents, GSIs have been repurposed as anticancer agents due to their anti-Notch activity (Figure 2). During the phase I study of PF-03084014 (nirogacestat), impressive activity was observed against DT, with five out of seven patients (71%) experiencing partial response and the other two (29%) having stable disease [76], supporting a potential role of GSIs in the medical therapy of refractory DT. In a follow-up study, of 16 evaluable patients, five (29%) experienced a confirmed partial response and were on study for more than two years and other five (29%) had prolonged stable disease as their best response. Importantly, nirogacestat was well tolerated [77].

A phase 3 trial comparing nirogacestat to placebo in adult patients with DT was recently opened and is expected to confirm the promising results of the previous studies [NCT03785964].

**Wnt/ β-catenin inhibitors**. Given the central role of the Wnt and β-catenin pathway in DT formation, their inhibitors were hypothesized as novel therapeutic agents that could be active in DT. Tegatrabetan (tegavivint, BC-2059) directly and selectively interferes with the interaction between β-catenin and transducin β-like protein 1 (TBL1) and TBL receptor 1 (TBLR1). Disruption of β-catenin-TBL1/TBLR1 binding inhibits β-catenin nuclear translocation and promotes its degradation (Figure 1). Activity of the molecule has been shown in vitro and in vivo in a chemotherapy-resistant metastatic osteosarcoma model [78]. A first-in-human phase I clinical trial is currently recruiting DT patients [NCT03459469].

**Immune checkpoint inhibitors**. Recent studies suggest that DT are characterized by a peculiar immune infiltration without expression of PD-L1 [22]. Whether these immune cells could be triggered against the DT cells is still under investigation; in particular the activity of the combination of nivolumab (an antibody targeting programmed cell death protein 1, PD-1) and ipilimumab (an antibody targeting cytotoxic T-lymphocyte-associated protein 4, CTLA-4) is being investigated in a large multicohort trial recruiting patients affected by rare cancers, including DT [NCT02834013].

## 6. Conclusions

In the last decades, the therapy of DT has radically changed, moving from an aggressive surgical approach to a more conservative one. The proven efficacy of low-dose chemotherapy and—hopefully and even more so—TKIs and other targeted agents will further confirm the prominent role of medical therapy in the management of DT.

As this scenario unfolds with novel clinical trials, a number of questions need to be addressed. Among these, the most pressing are: identifying which therapy has higher rates of partial responses to select the best drug in symptomatic cases requiring rapid disease shrinkage; exploring the best sequences of medical treatments to assess whether one sensitizes to—or vice versa reduces—the benefits of the following; understanding whether genetic, molecular or clinical factors may predict the best treatment for each patient. Additional studies to understand whether the combination of active agents may be more effective than their sequence will also be needed.

In this dynamic situation, it must be stressed that patients affected by DT globally already have a very long outcome, with survival times of decades after diagnosis. In this population, quality of life is a crucially important endpoint that needs to be taken in account when selecting the treatment of choice or designing clinical trials. Efforts to identify and assess quality of life issues in DT patients are therefore needed [79]. In this respect, patients advocacy groups—besides their classical role in providing patients with information and support—are of particular importance, as they can help doctors and researchers in defining adequate and relevant patient-reported outcomes to be included in studies; in designing clinical trial through definition of endpoints relevant to patients; in supporting patients’ accrual in clinical studies.
